# Analyzing a co-occurrence gene-interaction network to identify disease-gene association

**DOI:** 10.1186/s12859-019-2634-7

**Published:** 2019-02-08

**Authors:** Amira Al-Aamri, Kamal Taha, Yousof Al-Hammadi, Maher Maalouf, Dirar Homouz

**Affiliations:** 1 Department of Electrical and Computer Engineering, Abu Dhabi, United Arab Emirates; 2 Department of Industrial and Systems Engineering, Abu Dhabi, United Arab Emirates; 3 Department of Physics, Khalifa University of Science and Technology, Abu Dhabi, P.O. Box 127788, United Arab Emirates

**Keywords:** Text mining, Disease-gene association, Biological NLP, Biomedical literature, Genetic network

## Abstract

**Background:**

Understanding the genetic networks and their role in chronic diseases (e.g., cancer) is one of the important objectives of biological researchers. In this work, we present a text mining system that constructs a gene-gene-interaction network for the entire human genome and then performs network analysis to identify disease-related genes. We recognize the interacting genes based on their co-occurrence frequency within the biomedical literature and by employing linear and non-linear rare-event classification models. We analyze the constructed network of genes by using different network centrality measures to decide on the importance of each gene. Specifically, we apply betweenness, closeness, eigenvector, and degree centrality metrics to rank the central genes of the network and to identify possible cancer-related genes.

**Results:**

We evaluated the top 15 ranked genes for different cancer types (i.e., Prostate, Breast, and Lung Cancer). The average precisions for identifying breast, prostate, and lung cancer genes vary between 80-100%. On a prostate case study, the system predicted an average of 80% prostate-related genes.

**Conclusions:**

The results show that our system has the potential for improving the prediction accuracy of identifying gene-gene interaction and disease-gene associations. We also conduct a prostate cancer case study by using the threshold property in logistic regression, and we compare our approach with some of the state-of-the-art methods.

**Electronic supplementary material:**

The online version of this article (10.1186/s12859-019-2634-7) contains supplementary material, which is available to authorized users.

## Background

**Fig. 1:**
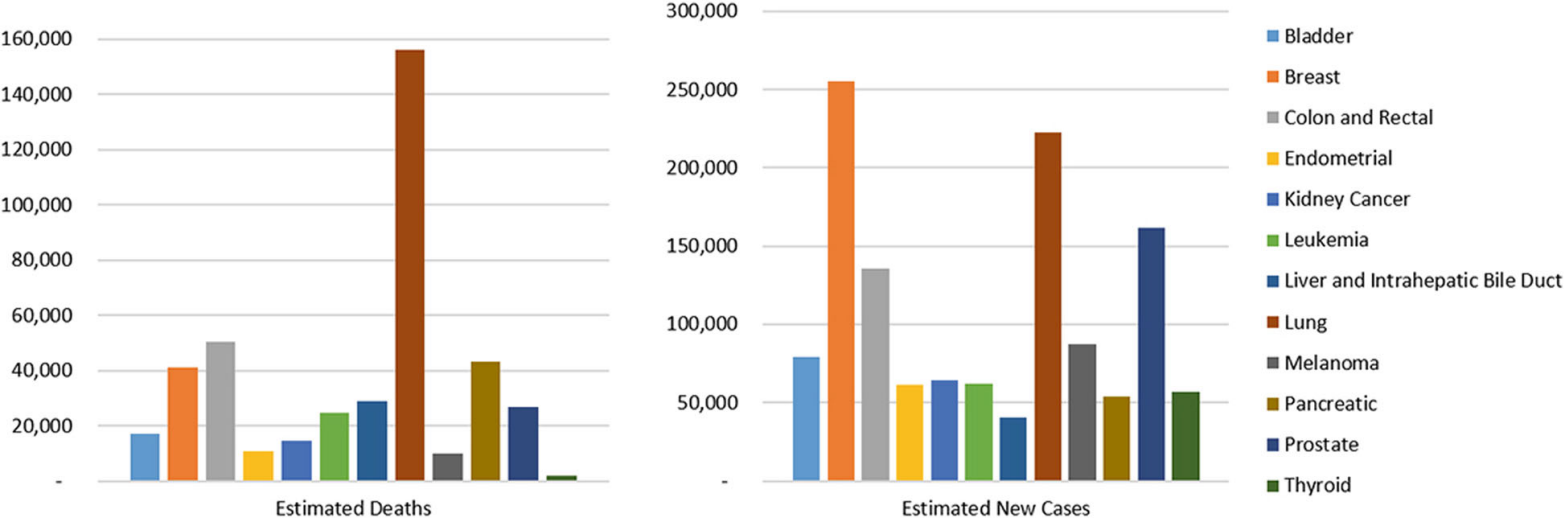
Number of new cases and deaths for each common cancer type from NIH [2]

According to NCCDPHP (National Center for Chronic Disease Prevention and Health Promotion), cancer is among the top 10 causes of deaths for 2014 in the United States [1]. Cancer affected about 8.8 million deaths in 2015 worldwide, with Lung cancer being the leading cancer cause of death according to the World Health Organization. The National Institutes of Health (NIH) in association with the American Cancer Society (ACS) reported the common cancer types in 2016 [2, 3], which is illustrated in Fig. 1. There are many efforts directed towards the treatment of this chronic disease, but the most important direction for more effective treatments starts with enhancing the understanding of cancer and the roots of its cause.

Cancer is a disease that is partially genetic, and the reason behind many genetic diseases and disorders is mutated genes. Mutations in genes lead to harmful consequences and genetic diseases [4]. Genes generally code for proteins. A single protein holds the responsibility of many functions within the cell. Thus, genetic mutations would lead to the creation of nonfunctional proteins. For instance, for genes coding for proteins involved in cell division, a mutation will interrupt the normal process of cell proliferation and death [5]. Genes that control cell division and growth are usually referred to as Tumor suppressor genes. Any alteration or mutation to these genes will disrupt the normal cell division process resulting in cell division over-activation, and will eventually lead to the development of a tumor (cancer).

Since abnormal proteins functions are highly associated with the occurrence of cancer, a large number of cancer studies focus on protein/gene functions. Such studies provide the critical knowledge needed in designing cancer diagnosis and treatment interventions. Over the two past decades, a large body of bioinformatics research was directed towards protein function predictions (PFP). Bioinformatics researchers focused their efforts on developing computational methods that assign and interpret the functions of proteins.

The PFP techniques are varied depending on the source of information (i.e., sequence-based, structure-based, text mining, and protein-protein interactions). These methods also influenced disease-gene association studies and disease gene prediction [6]. In general, the huge growth in biological data influenced a similar evolution in the biomedical literature. A huge effort in bioinformatics is directed towards the use of the rapidly growing biomedical literature to infer the disease-related genes by extracting this information directly from the text [7, 8]. The biomedical text mining approaches also referred to as BioNLP approaches, employ different Natural Language processing (NLP) techniques to extract descriptive information on biological entities and disease.

In this paper, we propose a simple yet powerful disease-gene association identification method based on analyzing a co-occurrence genetic network. We combine the information extraction method with rare event classification and then perform network analysis. We first construct a gene-gene-interaction network based on the number of times the genes and their Gene Ontology (GO) terms appear in the PubMed articles. We extract several features from the text to represent each pair of genes in a vector of variables. We employ two rare-event classification models to optimize the prediction accuracy and to consider the rareness of possible positive gene connections. We trained our data with linear and non-linear classifiers, and we present the results obtained for each one. Following the prediction of gene-gene interactions, a subnetwork is extracted to represent the disease-related network. We then use a network analysis tool to identify the network parameters, properties, and centrality measures. We use the centrality measure scores to rank the top *n* genes and evaluate them using a disease-gene association benchmark. In this work, we evaluate our approach for three cancer types (i.e., Prostate, Breast, and Lung).

We provide a demo that outputs the set of genes that are related to an input gene from the gene-gene-interaction network that the system has constructed. The demo also provides the list of related genes for three cancer types mentioned in this work by allowing the user to choose either classification models. The last option is to view the gene-gene-interaction networks constructed by a software for network analysis and visualization. The demo is available at:

- http://ecesrvr.kustar.ac.ae:8080/humangene/index.html.

## Related work

A well-known way to study proteins is through identifying similar proteins that interact with each other. A typical feature of proteins is the fact that they don’t work alone. Proteins interact or bind with each other to carry through a certain function [9]. Predicting the protein/gene interactions at their abstract level for the whole genome (i.e., the human genome, the yeast genome, etc.) results in constructing genetic interaction networks. Several approaches use previously known knowledge about the protein/gene to construct PPIs/GGIs. Among these various approaches, many have used the information within the biomedical articles to accomplish this goal. Although various literature analysis approaches have been presented in the past decade, the rapid growth of the biomedical publications encourages the continuous development of methods that automatically extract the information presented in the biomedical articles.

Studying the genes or proteins functions has proven to have a direct link to the detection of disease and the discovery of drugs. A missing or mutated protein in the cell is responsible for the cause of a disease. Therefore, the study of disease-gene association (DGA) has been widely conducted, especially in the field of biomedical literature mining. Similarly to the basic text mining approaches, DGA approaches can take a simple or a complex direction. In general, a relation extraction algorithm needs to be implemented in order to use the biomedical literature to find genes related to a certain disease [10]. Extracting DGA could depend on the mentions of both the disease and the genes, or analysis of already constructed genetic networks. Network analysis method is used in many text mining approaches [11–13]. One of the earliest approaches that extract disease-gene association based on text mining techniques and network analysis is proposed by Özgür et al. [14]. This method starts with the assumption that the central genes in their constructed disease genetic network are highly associated with the disease. After the gene-gene-interaction network is constructed, centrality measures are applied to rank the top genes in the network that are more likely to be associated with the target disease (i.e., Prostate Cancer). Another very similar approach by Quan & Ren targets the study towards Breast Cancer [15]. It also applies centrality measures to analyze the constructed network, but the difference is in the technique followed for building the network. Quan & Ren select only important sentences that include interaction verbs between genes or diseases.

There are much simpler approaches that depend only on the co-occurrence frequency among biological entities (genes, proteins, and diseases) [16]. GO terms are proven to improve the overall performance of the DGA approaches like in [17]. This application applies proximity relation between genes and diseases mentioned in the biomedical text, while also identifying the GO terms annotating the genes and diseases (calculate the semantic similarity). Another approach by Sun et al. uses GO annotations as one source for predicting disease-gene associations [18]. BioNLP has been engaged in many disease/Network-based prediction algorithms, and that is shown in details in the review study by Zhu et al. [19]. Including several Natural Language Processing techniques in the development of these applications can make a complex system. However, using NLP with text mining has shown to perform more efficiently to extract relevant information [20]. Some researchers focus on the identification of disease-related genes without predicting new candidate genes like in DigSee [21]. This application is a search engine that finds and highlights the associations among Cancer genes.

In this paper, we tackle some of the limitations that the above studies have by first identifying the GO terms in the abstract text along with the gene name rather than calculating the GO terms semantic similarities between the genes or diseases mentioned in the text. Also, we extract features at three levels of text (i.e. abstract, sentence, and semantic), rather than limiting the search for interacting genes in the sentences or abstracts only. One of the key contributions of this work is to utilize rare-event classification which has many advantages over other classification methods. With this classification method, we can use small datasets to train and test the classifier [22–24]. To the best of our knowledge, this is the first work that utilizes rare-event classification with the use of biomedical text mining approach. Recognizing the sparsity of biomedical data when designing a text mining prediction system is crucial since the possible negative connections between genes outnumber the possible positive connections. We also use the threshold property of the classifier to rank the predicted genes which presents novel observations.

## Methods

In this section, we explain the process of constructing the co-occurrence genetic network for the human genome (“Co-occurrence network” section). Our research focus is on using the GO terms as biological terms to help with the information extraction step. We also present linear and non-linear rare-event classifiers. In “Disease-gene association” section, we then describe the process of extracting disease-gene associations based on network analysis.

### Co-occurrence network

Constructing the co-occurrence genetic network consists of the following main steps:

#### Information extraction

We used UniProtKB/SwissProt [25] to download the primary/official list of genes in order to build the gene-gene-interaction network. We downloaded a total set of 20,183 human genes. In this work, we also identify the Gene Ontology (GO) terms from the text. Gene Ontology is one of the most popular bio-ontology [26]. It annotates genes based on the three main functionalities of genes, i.e., cellular location, molecular function, and biological process. A gene is annotated by one or many GO terms and thus, GO terms are highly descriptive of the genes functionality. We downloaded the list of GO terms that are associated with each gene retrieved from UniProtKB/SwissProt using QuickGO [27]. Therefore our system mainly looks for the gene names and GO terms in the text of biomedical articles. Each gene in the list of genes should be annotated by at least one GO term and should also be mentioned in at least one PubMed article. As for the extraction text, we have used a set of PubMed abstracts retrieved from the National Center for Biotechnology Information (NCBI) [28]. We use abstracts as they are publicly available data and they usually hold the main outcomes of the biomedical experiments. We used the E-utilities provided at NCBI to search and download the abstract texts that mention at least one human gene. We used two main e-utilities that are "e-search" to search the PubMed IDs associated with a target gene, and "e-fetch" to retrieve and download the PubMed abstract text using the abstract ID from the previous e-utilities query. We retrieved a total of 7,894,920 abstracts in February 2017 and saved them into a local SQL database.

Our proposed system automatically extracts different features from the text based on co-occurrence the biological terms *“gene-gene" or “gene-GO term"*. In addition, the system looks for the co-occurrence frequency at three different levels of text (i.e., abstract level, sentence level, and semantic level). The abstract and sentence levels respectively indicate the number of times the two terms appear in the same abstract and the same sentence. The semantic level expresses the number of times the two terms appear to have a semantic relationship in the text. That is, the two terms show a positive relationship when we look closely at the sentence. Accordingly, we look for phrases which indicate that the biological terms are interacting or related to each other (e.g., “binds with", “interacts with", “and", “or", etc.). We study the semantic level to have a better understanding of the relation between two biological entities, specifically in the sense of inferring if they are related/connected to each other. The semantic level expresses the “semantic similarity” which is defined as the measure of resemblance between two biological entities.

We used the Java APIs provided by LingPipe [29] to develop name entity recognition. Through LingPipe, we identified biological entities (i.e., genes, and GO terms), developed sentences tagging, and word tokenization. Each abstract is parsed through LingPipe library. The features for each pair of genes is then extracted and analyzed by updating the occurrence status of each biological entity according to the three levels of text (i.e., abstract, sentence, semantic).

We represent each pair of genes by the previously extracted information in a vector of features. In the framework of this study, a pair of genes *X*_*i*_ is represented by nine features.

$$X_{i}=\left \langle W_{1},W_{2},W_{3},W_{4},W_{5},W_{6},W_{7},W_{8},W_{9} \right \rangle $$

Each feature measures the likeliness between the two genes in the pair. Also, Each feature will represent either the direct (*gene-gene*) or the indirect (*gene-Go term*) co-occurrences of the two genes. Since we keep track of the occurrence frequency of the biological terms at three levels of text, each feature will indicate a level (i.e., abstract, sentence, semantic). The way to compute each feature is by calculating the number of times the two biological terms are co-occurred over their individual appearance in the level of the text. Table 1 shows a description of the nine features for the pair of genes (*g*_1_,*g*_2_), with regards to the biological terms they are representing and the level of text they are targeting. The information extraction component will result in a table of vectors (pairs) referred to as Table(*X*), where *X*_*i*_ is a row in the table. Further details on this information extraction technique are introduced in a recent study [23].

**Table 1:** Description of features for the pair (*g*_1_,*g*_2_)

Feature	Biological terms	Text level
*W*_1_	*g*_1_ and *g*_2_	Abstract
*W*_2_	*g*_1_ and *g*_2_	Sentence
*W*_3_	*g*_1_ and *g*_2_	Semantic
*W*_4_	*g*_1_ and the GO terms of *g*_2_	Abstract
*W*_5_	*g*_1_ and the GO terms of *g*_2_	Sentence
*W*_6_	*g*_1_ and the GO terms of *g*_2_	Semantic
*W*_7_	*g*_2_ and the GO terms of *g*_1_	Abstract
*W*_8_	*g*_2_ and the GO terms of *g*_1_	Sentence
*W*_9_	*g*_2_ and the GO terms of *g*_1_	Semantic

Each feature measures the number of times the two biological terms are co-occurred over their individual appearance in the level of text

#### Rare-event classification:

The table of vectors (*X*) that is produced by the information extraction step is fed to a rare-event classification model. Due to the fact that the possible negative relations among genes (non-events) outnumber the possible positive relations (events), we chose to employ a rare-event classifier that will address the rarity of positive connections. In this work, we use a linear rare-event classifier (*Weighted Logistic Regression* (WLR) [22]), and we also employ a non-linear classifier alternative (*Weighted Kernel Logistic Regression* (WKLR) [30]). Both classifiers optimize the prediction accuracy and reflect the sparsity of the biomedical data by using a reasonable sample size [31]. The linear classifier (WLR) is particularly more effective than WKLR is terms of tuning the hyperparameters for large datasets. Moreover, WKLR could be slower than WLR since it represents the data in a high dimensional space. However, it can better capture the data behavior since it separates the data non-linearly [32].

We used a regularization parameter (*λ*) in both classifiers to avoid singularities and overfitting. Next, we provide a general description of the classifiers, and we list all their related equations in Table 2. In both models (WLR and WKLR), the vector of features is represented in a logit transformation function defined by Equation 4 for WLR and Equation 6 for WKLR. *p*_*i*_ is the probability of the pair of genes being interacting, *β* in Equation 4 is a vector of parameters that differentiate the events and the non-events (the positive class and the negative class). *α* in Equation 6 is the dual variable (vector) that also indicates the separation of events and non-events. *X*_*i*_ is a row in Table(*X*), and it is just the vector of features for a pair of genes. *k*_*i*_ also represents a pair of genes, but the difference is that WKLR transforms the data to a higher dimensional space, hence *k*_*i*_ is the *i*th row in the kernel matrix *k*(*X*_*i*_,*X*_*j*_)=*K* (see Eq.(5)). The kernel used in WKLR is the Gaussian Radial Basis Function (RBF) kernel [33] as shown in the equation below. *σ* is the kernel parameter that defines the width of the kernel. This parameter along with the regularization parameter (*λ*) are chosen from a range of values and are tuned using bootstrapping.

**Table 2:** The logit transformation and regularized log-likelihood for both classifiers (WLR and WKLR)

Model	Logit transformation	Regularized log-likelihood
WLR	$ {ln \left (\frac {p_{i}}{1-p_{i}} \right)=X_{i}\beta } \text {(1)}$	$ {lnL\left (\beta \right) = \sum \limits _{i=1}^{n} w_{i}ln\left (\frac {e^{y_{i}x_{i}\beta }}{1+e^{x_{i}\beta }} \right)- \frac {\lambda }{2}\left \| \beta \right \|^{2}} \text {(2)}$
WKLR	$ {ln\left (\frac {p_{i}}{1-p_{i}} \right) = k_{i}\alpha } \text {(3)}$	$ { lnL_{W}\left (\alpha \right) = \sum \limits _{i=1}^{n} w_{i}ln\left (\frac {e^{y_{i}k_{i}\alpha }}{1+e^{k_{i}\alpha }} \right)- \frac {\lambda }{2}\alpha ^{T}K\alpha } \text {(4)}$

The detailed description for each equation is reported in “Rare-event classification:” section

5 $$  {k \left(X_{i}, X_{j}\right) = e^{\left(-\frac{1}{2\sigma^{2}}\left\| x_{i}-x_{j} \right\| \right)^{2}}}  $$

The best *β* and *α* vectors are estimated by maximizing the log-likelihood. The difference between the two models is presented in estimating the log-likelihood where it is expressed in Eqs. 5 and 7. In both equations: *y*_*i*_ is 1 if the *i*th training example pair was related and 0 otherwise, *n* is the total number of training examples, and *λ* is the regularization parameter. The log-likelihood is adjusted using the weight *w*_*i*_ that represents the proportion of events to non-events. This weight introduces rare-event classification and reflects the imbalanced data problem.

#### Prediction:

We trained our system using STRING training dataset that provides the information of experimentally verified related genes [34]. Although STRING is a source for interacting genes/proteins based on experimental and computational methods, we only retrieved the experimentally verified interactions. Each pair of genes represented by the nine features (*recall* “Information extraction” *section*), is assigned the value “1" to indicate that the pair of genes is confirmed to be experimentally related according to STRING. We assigned the value “0" to pairs that do not appear to be related, but both genes have to be appearing in STRING experimentally verified interactions network.

We use **Bootstrapping** to train the classifiers and to adjust the regularization parameter (*λ*) and the kernel parameter (*σ*). Bootstrapping is a re-sampling method that allows the generation of a large number of samples over multiple rounds. It is a simple and effective technique for approximating the true error measure and for generating a confidence interval for the accuracy [35]. We evaluate the accuracy at each round and by tuning the parameters (*λ* and *σ*). The best accuracy is found by comparing all the accuracies obtained by the different values of the parameters. The best accuracy indicates that we found the best fit parameters *β* and *α* that will be used for the prediction.

For the WLR classifier, we found the best *β* vector at *λ*=4328, and we predict the relation for the pairs of genes using the following equation: *0.5 is the default threshold for prediction in logistic regression.*

6 $$  {y_{i}=\left\{\begin{array}{ll} 0, & \,\,\,P(y_{i}|X_{i}\beta)\leqslant 0.5\\ 1, & \,\,\,P(y_{i}|X_{i}\beta)> 0.5 \end{array}\right. }  $$

As for the WKLR classifier, the best *α* vector was found at *λ*=5.7×10^−3^ and *σ*=0.5, the relation prediction is evaluated using the following equation:

7 $$  {y_{i}=\left\{\begin{array}{ll} 0, & \,\,\,P(y_{i}|k_{i}\alpha)\leqslant 0.5\\ 1, & \,\,\,P(y_{i}|k_{i}\alpha)> 0.5 \end{array}\right. }  $$

**Fig. 2:**
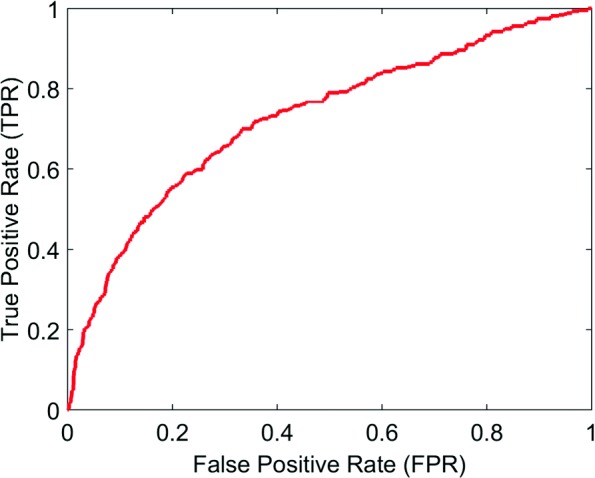
ROC curve for Training the data using WLR. TPR is increased at low FPR

**Fig. 3:**
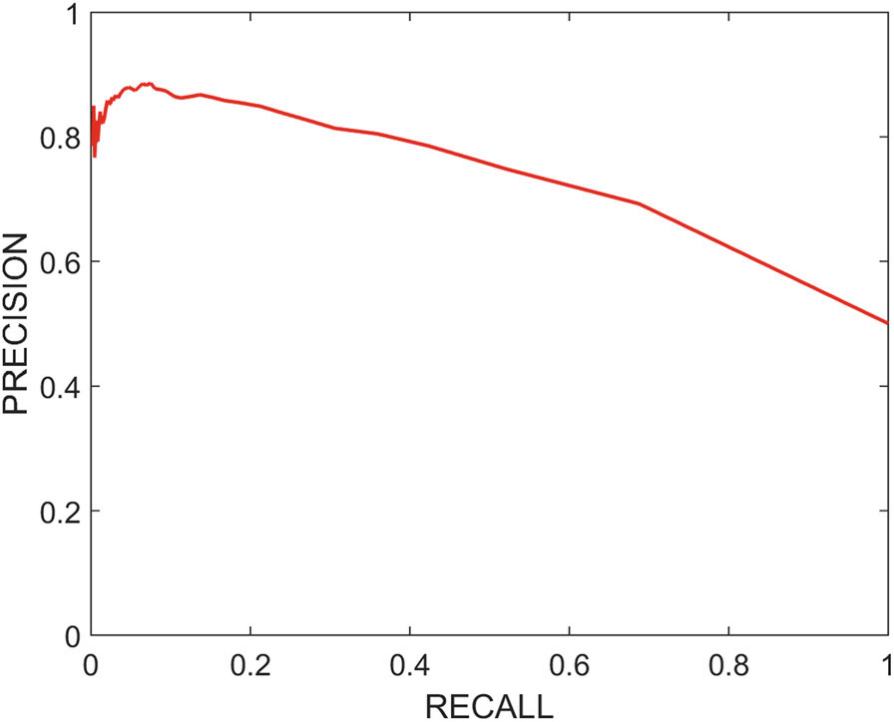
Precision-Recall Curve Using WLR

**Fig. 4:**
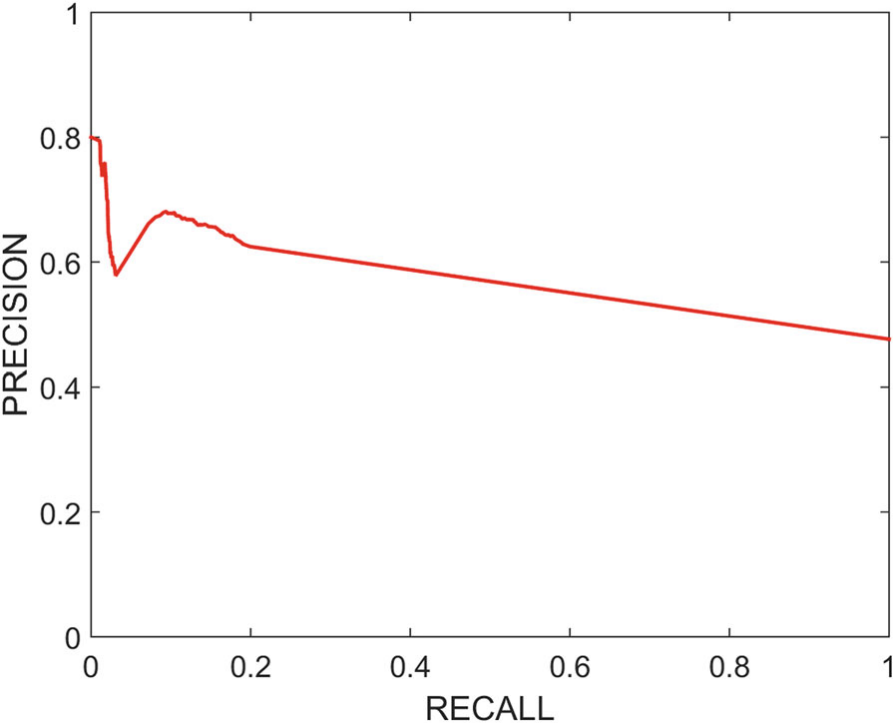
Precision-Recall Curve Using WKLR

We show the Receiver Operating Characteristic (ROC) curve in Fig. 2 to assess the quality of our system. ROC curve is a plot of the true positive rate (TPR) against false positive rate (FPR) at different thresholds. We also computed the Area Under the ROC Curve (AUC) measure in Tables 3 and 4 to show how well our system can separate the connected and unconnected genes using WLR and WKLR respectively. With WKLR, we achieved higher accuracy than WLR for both classes as seen in Table 4. In Figs. 3 and 4, we show how our system balances both recall and precision by identifying the performance measures (true positives, false positives, etc.) according to STRING, and by using WLR and WKLR.

**Table 3:** Accuracy measures from training a data of pairs of genes using WLR

	Accuracy	AUC
Class 0 (unrelated)	68	74
Class 1 (related)	68

**Table 4:** Accuracy measures from training a data of pairs of genes using WKLR

	Accuracy	AUC
Class 0 (unrelated)	71	78
Class 1 (related)	85	

Using either classifier, we can predict the interacting genes and, hence, construct the human gene-gene-interaction network. In the next section, we describe the process of identifying disease-related genes using network analysis.

### Disease-gene association

In “Co-occurrence network” section, we constructed the genetic co-occurrence network for the entire human genome. We are going to use this network to extract disease-related subnetworks. In this work, we are focusing the study on different Cancer types (i.e., Breast, Prostate, Lung, etc.). As shown in Fig. 5, we follow a process of steps to construct disease subnetworks, analyze these networks and identify new candidate genes that could be linked directly to the disease. The steps are as follows:

1. **Initial list of seed genes:** The process of constructing the disease subnetwork starts with retrieving the genes related to the disease under consideration according to a high-quality reference source. We used Online Mendelian Inheritance in Man (OMIM) to download the seed genes that we are going to use to build the subnetwork [36]. OMIM is a comprehensive collection of human genes and diseases that is being updated daily and publicly available. Moreover, it is commonly used in most of the methods that identify disease-gene associations. OMIM provides the access to its database through an API. The OMIM API URLs consists of handlers, parameters and a unique API key that is given upon request to the user. We used the ’geneMap’ handler to search and retrieve all the data related to a certain disease entry.

**Fig. 5:**
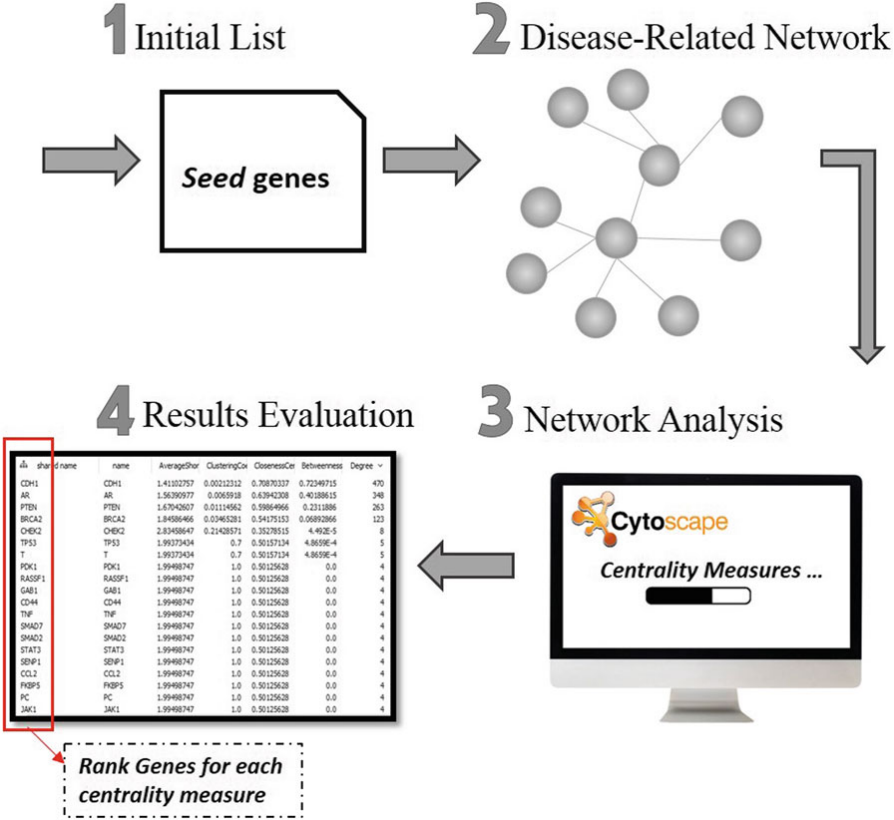
The process of network analysis and disease-gene identification
2. **Building disease-related subnetwork:** Using the seed genes as a start for building the network, we retrieved from our previously predicted network all the genes that are related to at least one seed gene. All the pairs in the generated subnetwork include at least one seed gene. The subnetwork is then analyzed to get further candidate genes that could be directly related to the disease of study. The list of related genes for the three cancer networks (Breast, Prostate and Lung Cancer) by using either WLR as a classifier or by using WKLR as a classifier are available via the demo link provided in “Background” section.
3. **Network Analysis (centrality measures):** We used **Cytoscape** network analyzer to perform the analysis for the constructed subnetwork. Cytoscape is an open-source visualization tool that offers interactive network analysis [37]. It computes the network parameters such as the number of nodes and edges, and it reports several properties of the network such as the network flow. Cytoscape computes different centrality measures to rank all the genes in the network and identify the most relevant to the disease. Centrality measures identify how important each node is and how does it affect the network. In this work, we applied several centrality measures, and each is defined below:
  - *Degree centrality*
    The degree of a node is the number of nodes that are connected to it. Alternatively stated, it is the number of edges adjacent to the node as well. The degree centrality indicates the popularity of the node, hence, the more neighbors a node has, the more important the node is.
  - *Eigenvector centrality*
    This centrality measures the extent of effect a node has in a network. Similarly to the degree centrality, the eigenvector centrality scores the number of neighbors of a node. However, the difference is that the neighbors, in this case, are only considered if they have the characteristics of being high quality or high scoring nodes. A node will score a high eigenvector value if it is also connected to nodes with high eigenvector values. Based on this, the node centrality is dependent on the quantity and the quality of its connections. A node is said to be well-connected if it has more prestigious nodes connected to it.
  - *Closeness centrality*
    This centrality is a measure of how close a node is to all other nodes in the network. A node with a high closeness value is of interest, as it implies that the node is closer to the center of the network. It also implies that the node has a high effect on the nodes surrounding it. Closeness centrality is computed by calculating the inverse of the sum of the shortest distances between each node and every other node in the network. It can be simply put that higher closeness means a smaller total distance of a node to the other nodes.
  - *Betweenness centrality*
    Betweenness indicates the extent to which a node affects the flow of data within the network. It measures the number of times a node serves as a channel in the shortest paths between two other nodes. The higher the betweenness value is, the more important the node is in controlling the network connections. Betweenness is computed by calculating the number of shortest paths between other nodes passing over this node.
4. **Results Evaluation:** All the previous centrality measures give us a summary of the network properties, by reporting a score for each node (gene) in the network. In order to test the prediction quality of our method, we ranked the genes based on their score values with each of the described standard centrality measures. That is, for each centrality measure we evaluated the top 15, 25, 45, etc. genes by using different benchmarks that hold already known disease genes. The tests and results validation are reported in the next section.

## Experimental results

We implemented this system in Java, and we run it on Intel(R) Core i7 processor, with a CPU of 3.4 GHz and 16GB RAM, under Windows10. We used Ling Pipe APIs for the information extraction algorithm and implemented the classification model in MATLAB. We determine the interactions among human genes based on their frequency in the biomedical texts.

The co-occurrence network generated by our system is analyzed to identify disease-gene associations. More specifically, we study cancer-related genes found in the co-occurrence network. We followed the steps mentioned in “Disease-gene association” section to analyze the co-occurrence genetic network. We first retrieve an initial list of genes associated with the target cancer type, using OMIM database. We then build a cancer-related subnetwork using the already generated co-occurrence network. We establish the subnetwork through a search for genes that interact with at least one seed gene. In this study, we construct subnetworks for three different types of Cancer (i.e., Prostate, Breast, and Lung). We gathered 18 prostate cancer seed genes, 23 for breast cancer, and 16 for lung cancer. Table 5 lists the seed genes compiled for each cancer type. It has not escaped our notice that OMIM does not include *“BRCA1 gene"* in the list of breast cancer genes (MIM number: 114480). However, this gene appears to be associated with breast-ovarian cancer syndrome (Mim number: 604370). We did not manually include *BRCA1* in the list of breast cancer genes for the sake of source data integrity. Using the seed genes to construct the disease-related network, we counted the predicted interactions for the three cancer types. These interactions are generated for the two classifiers used in this study (WLR and WKLR). We included the Network images for each cancer type via the demo link provided in “Background” section. We also show Cytoscape’s report on the subnetwork’s parameters such as the network diameter, clustering coefficient, number of interactions and number of nodes in Table 6.

**Table 5:** The seed genes retrieved from OMIM

Prostate	Breast	Lung
PCAP	RAD54L	FASLG
HPC5	CASP8	CASP8
MAD1L1	BARD1	DLEC1
HPC4	PIK3CA	RASSF1
HIP1	HMMR	PIK3CA
MSR1	NQO2	IRF1
KLF6	ESR1	PRKN
PTEN	RB1CC1	EGFR
MXI1	SLC22A1L	BRAF
CD82	TSG101	MAP3K8
BRCA2	ATM	ERCC6
CDH1	KRAS	SLC22A1L
ZFHX3	BRCA2	PPP2R1B
HPCQTL19	XRCC3	KRAS
HPC3	AKT1	ERBB2
CHEK2	RAD51A	CYP2A6
HPC6	PALB2	
AR	CDH1	
	TP53	
	PHB	
	PPM1D	
	BRIP1	
	CHEK2

**Table 6:** The Cancer-related gene-interaction networks properties as reported by Cytoscape

	Diameter	Nodes	cc ^∗^	Interactions
Prostate				
WLR	9	257	0.038	275
WKLR	6	1808	0.086	2479
Breast				
WLR	8	504	0.103	693
WKLR	6	3126	0.161	5986
Lung				
WLR	7	555	0.070	691
WKLR	6	2355	0.067	3959

^∗^ cc refers to clustering coefficient

We used Cytoscape to analyze the networks using closeness, betweenness, degree and eigenvector standard centrality measures. Each measure produces a list of genes (nodes in the network) that are ranked by the centrality score. We evaluate the quality of our system in identifying disease-related genes with reference to two benchmarks:

a ***MalaCards*** [38]:
  MalaCards is a database of human diseases, their related-genes annotations, and the database is affiliated with GeneCards [38]. It holds about 20,000 disease entries integrated from more than 70 data sources. In a study by Rappaport et al. MalaCards is shown to outnumber OMIM and UniProt in the average number of disease-gene associations [39]. In this experiment, we retrieved from MalaCards the gene-disease associations that are marked as “elite" genes. An elite gene in the framework of MalaCards is defined to be that from sources that are manually curated and contains strong and reliable association to the disease.
b ***NCI’s GDC*** [40]:
  NCI’s GDC is short for the National Cancer Institute’s Genomic Data Commons. It is a data portal that holds a collection of descriptive information on cancer genomics. It is part of the National Institutes of Health (NIH), which is a research agency governed by the U.S. Department of Health and Human Services. We retrieved from the GDC portal cancer-related genes that are marked as being part of the Cancer Gene Census (CGC), which is an ongoing effort to categorize genes involved directly to cancer [41].

For each centrality measure, we evaluated the top 15 ranked genes. In general, the top *n* ranked genes have the highest centrality scores. Particularly, as *n* increases the centrality scores decrease and sometimes approach 0, which means that it is less likely to find genes related to cancer as *n* increases. We show the effect of centrality scores on the percentage of related genes using MalaCards as a benchmark. In Table 7, we report the precisions of all centrality measures for the top *n* ranked genes related to Lung Cancer where the pairs in the lung-cancer-subnetwork were predicted using WLR. The percentages of the top *n* genes start off with high values of up to 99% performed by eigenvector. As *n* increases though, the precisions go down for the four centrality measures, and they converge to each other.

**Table 7:** Percentage of top *n* genes related to lung cancer based on MalaCards database

Top *n*	Closeness	Betweenness	Degree	Eigenvector
10	80.00	80.00	90.00	99.00
15	73.30	80.00	86.70	93.30
20	70.00	70.00	90.00	90.00
30	60.00	70.00	83.33	76.67
50	48.00	56.00	72.00	72.00
75	40.00	48.00	54.67	58.67
100	36.00	50.00	50.00	52.00
125	31.20	43.20	43.20	47.19
225	20.44	28.44	28.44	29.77
300	17.33	22.33	22.33	24.33
450	17.11	17.11	17.11	17.33
500	15.60	15.60	15.60	16.20
555	15.31	15.31	15.31	15.31

In the following test, we evaluated the performance of the system in identifying the genes associated with each cancer type, using two benchmarks: MalaCards and NCI’s GDC. For this test, we enumerated the top 15 genes ranked with each centrality measure and tested their precision. Tables 8 and 9 show the percentage values for the three diseases against the two chosen benchmarks, and the results are discussed below. We included the datasets of the two benchmarks for each cancer type in the supported files [see Additional file 1].

**Table 8:** The precision measures of the top 15 genes by each centrality measure and against MalaCards

	Closeness	Betweenness	Degree	Eigenvector
Prostate				
WLR	53.3	*86.7*	80	66.7
WKLR	46.7	80	*86.7*	66.7
Breast				
WLR	80	86.7	*93.3*	*93.3*
WKLR	46.7	*100*	*100*	86.7
Lung				
WLR	73.3	80	86.7	*93.3*
WKLR	60	*86.7*	*86.7*	*86.7*

The highest precisions are italic

**Table 9:** The precision measures of the top 15 genes by each centrality measure and against GDC

	Closeness	Betweenness	Degree	Eigenvector
Prostate				
WLR	*80*	60	66.7	*80*
WKLR	33.3	*60*	*60*	*60*
Breast				
WLR	73.3	40	53.3	*86.7*
WKLR	46.7	66.7	66.7	*80*
Lung				
WLR	20	20	33.3	*86.7*
WKLR	40	40	40	*60*

The highest precisions are italic

## Discussion

### Using MalaCards:

As can be seen from Table 8, degree centrality achieves the highest precisions in most of the models (WLR and WKLR) and cancer types. Betweenness and eigenvector centrality are second to degree centrality in terms of performance, as they achieve an average precision score of 86.86% and 82.23% respectively, where the highest precision is 100%, and the lowest is evaluated to 80%. The precision achieved by closeness centrality is the lowest across all models (average precision of 60%). Regarding the top 15 breast-cancer-genes predicted by WKLR model, the achieved precisions by betweenness and eigenvector show that all 15 predicted genes are considered associated to breast cancer with reference to MalaCards (Both precisions are 100%). To analyze the centrality precisions based on the classifier models, we noticed that in overall, WLR performs slightly higher than WKLR as the latter model tends to hold more interactions in the cancer-related genes subnetwork (number of interactions are reported in Table 6). Comparing the cancer types, breast cancer results show that our model(s) predicted most of the breast cancer genes according to MalaCards.

### Using NCI’s GDC:

Table 9 show the precision results for four centrality measures evaluated against NCI’s GDC Data. Eigenvector centrality achieves the highest precisions for all cancer types (average precision is 75.57%), with the highest value being evaluated 86.7% and the lowest to 60% which is considerably higher than most scores by other centrality measures. Betweenness and closeness centrality perform relatively worse with average precisions of 47.8% and 48.9%. With GDC, WKLR achieves higher average precision than WLR with both breast-related and lung-related genes. Out of the three cancer types, WLR predicts correctly 80% of prostate-related genes using both closeness and eigenvector centrality. With both benchmarks: MalaCards and GDC, the proposed system predicted correctly most genes using degree and eigenvector centrality.

### Combining MalaCards and NCI’s GDC:

Table 10 shows the precision results for the four centrality measures evaluated against both MalaCards and NCI’s GDC Data. As can be seen from the table, the precisions are improved extremely compared to the results in both Tables 8 and 9. One noticeable improvement is that except for closeness, all other centrality measures scored above 86% with all cancer types and all classification methods. The precision scores are also seen to be almost consistent for each cancer type. Lung cancer average precision results are the most improved among the cancer types when compared to the results by each dataset individually. Although closeness measures achieved the lowest average precision, the lowest precision is at 53.3%. Combining the two datasets assists in giving more of an accurate presentation of our system’s performance.

**Table 10:** The precision measures of the top 15 genes by each centrality measure and against both GDC and MalaCards

	Closeness	Betweenness	Degree	Eigenvector
Prostate				
WLR	93.3	93.3	93.3	86.7
WKLR	60	86.7	93.3	80
Breast				
WLR	80	86.7	93.3	93.3
WKLR	53.3	100	100	86.7
Lung				
WLR	73.3	80	86.7	100
WKLR	66.67	86.7	86.7	93.3

### The recall of seed genes:

We also evaluated the system in terms of recall performance measures. We report the percentage of initial seed genes that are retrieved among the predicted pairs from the whole human genome network *(recall* “Co-occurrence network” *section)*. This is an indication of the original coverage of the system’s predictions or connections in the co-occurrence network. The recall measure is computed by dividing the number of seed genes found in the co-occurrence network over the total number of seed genes which are 16, 18 and 23 genes respectively for lung, prostate and breast cancers. The recall scores are shown in Table 11. Both WLR and WKLR perform almost equally in this test. All the breast and lung cancer seed genes were already predicted and found in the co-occurrence network. About 66.6% (12 out of 18) prostate seed genes were found in the co-occurrence network using WLR classifier. By using WKLR classifier, about 72.2% (13 out of 18) prostate seed genes were found in the co-occurrence network.

**Table 11:** The recall of seed genes in the whole human genome network created by using either WLR or WKLR

Prostate seeds	Recall	Breast seeds	Recall	Lung seeds	Recall
WLR	66.6	WLR	100	WLR	100
WKLR	72.2	WKLR	100	WKLR	100

### An Example of Breast-Cancer candidate genes:

In this section, we aim at presenting breast-cancer related genes that are uniquely predicted by our proposed system. These genes are validated by MalaCards and NCI’s GDC. To the best of our knowledge, our system is the first to associate these genes with breast-cancer. We take the relatively recent proposed system by Quan & Ren [15] as a sample of the systems that miss to predict these genes. Table 12 shows the 30 top-ranked breast-cancer related lists of genes predicted by our proposed system and Quan & Ren. As the table shows, our uniquely predicted genes are not included in the list predicted by Quan & Ren. 83.3% of the genes predicted by our system and shown in Table 12 are validated by MalaCards and NCI’s GDC. These genes are marked with ’YES’ in the table. 70 present of our predicted genes shown in the table are seed genes and marked with ’Seed’. As Table 12 shows, there are four common genes predicted by both, our system and Quan & Ren. We consider the remaining genes predicted by our system (i.e., the genes that are not validated by MalaCards and NCI’s GDC) as ***“candidate genes”***. These genes need to be validated by experts. We will investigate them in a future work. Since the datasets used by our system and Quan & Ren are different, we did not evaluate the genes predicted by Quan & Ren against MalaCards and NCI’s GDC. The goal here is to show that our proposed system provides uniquely discovered genes.

**Table 12:** To the left, the Top 30 genes predicted by our system and their relevance to breast-cancer

Propsed system	Relevant	*Quan & Ren* [15]
BRCA2	YES+Seed	TNF
ESR1	YES+Seed	EGFR
CDH1	YES+Seed	CRC
BRCA1	YES	PTEN
PPM1D	YES+Seed	IL-6
NQO2	YES+Seed	AR
XRCC3	YES+Seed	BRCA1
TSG101	YES+Seed	EGF
CDKN2A	candidate	GAPDH
PALB2	YES+Seed	HR
BRIP1	YES+Seed	AML
PIK3CA	YES+Seed	CD4
MRE11A	candidate	STAT3
RAD54L	YES+Seed	AD
ERBB2	YES	MMP-9
CHEK2	YES+Seed	MS
RAD51C	candidate	RD
AKT1	YES+Seed	MYC
TP53	YES+Seed	S6
RB1CC1	YES+Seed	TP53
RB1	YES	ATM
HMMR	YES+Seed	IL-8
STK11	YES	AP1
BARD1	YES+Seed	MMP-2
RAD51	YES	GC
KRAS	YES+Seed	FBS
RAD50	candidate	ES
ATM	YES+Seed	RA
BACH1	Seed	CXCR4
CASP8	YES+Seed	BRCA2

To the right, a list of the Top 30 genes predicted by *Quan & Ren*

## Prostate cancer case study and comparison

We present in this section the Prostate Cancer Case Study in which we will compare our system with recent approaches. In order to conduct the comparison, we used the same datasets used in the other approaches and we re-constructed the co-occurrence network. The steps 1-4 are the pre-steps for the comparison (step 5):

1. **Seed genes:** We downloaded the initial list of genes that are related to prostate cancer using the gene/phenotype map in OMIM. We used this list to build the co-occurrence interaction network for prostate cancer.
2. **Downloading PMC articles:** We used PMC which is an electronic catalog of full-text PubMed articles. It offers free access to view and to download the articles via an FTP service. We downloaded all the PubMed articles that are associated with prostate cancer.
3. **Threshold Ranking:** In this experiment, we use the threshold property in our chosen classifiers (WLR and WKLR). As stated previously in Eqs. 6 and 7, 0.5 is the default threshold for prediction in logistic regression. A typical binary weighted logistic regression plot with a threshold of 0.5 is illustrated in Fig. 6. A perfect scenario would have the positive connections plotted to the right of the y-axis, and the negative connections plotted the left. However, this is not always the case as some positive and negative connections might overlap during the prediction process. In this test, we predict the relation among genes using different thresholds (i.e., 0.5, 0.6, 0.7 and 0.8) as seen in Fig. 6. As the threshold increases, the prediction line is moved away from the y-axis, which indicates stronger positive relations. We observed the pair of genes that keep on appearing at the different thresholds to effectively retrieve related genes (positive relations).

**Fig. 6:**
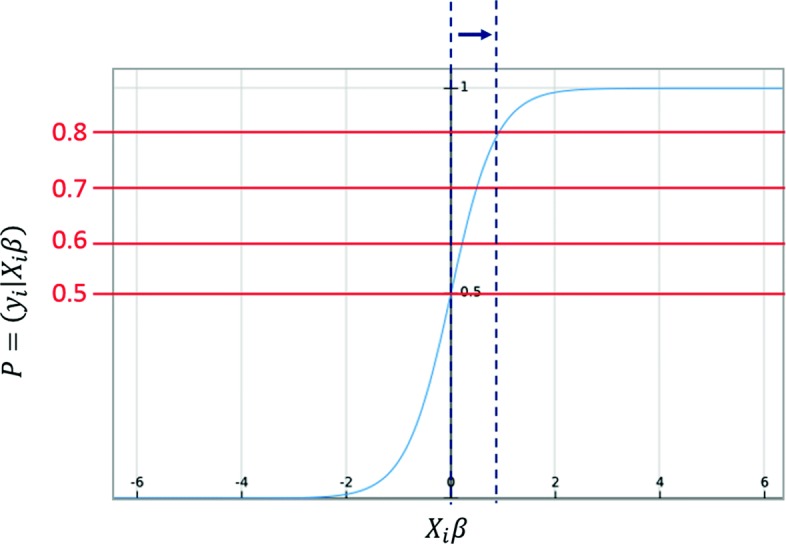
The prediction is made over several thresholds. As the threshold increases, fewer pairs are assigned to the positive class
4. **Comparison with recent approaches:** We evaluated our approach with CGDA [14], EDC-EDC [42] and MCforGN [43]. To compare to these approaches, we used the same ground truth data they follow (i.e., PGDB [44]). PGDB stands for Prostate Gene DataBase. It is a curated database of prostate related genes in general, and genes involved in prostate diseases.
  CGDA [14]: CGDA identifies disease-gene associations by analyzing the disease-related network. It builds the network by extracting the information on interacting genes from the biomedical literature. It then employs centrality measures to rank and identify disease-related genes.
  EDC-EDC [42]: EDC infers disease-gene association by extracting this information from the biomedical text. It proposes novel linguistic computational techniques to extract genes interactions. It employs a hybrid constituency–dependency parser for developing a biological NLP information extraction.
  MCforGN [43]: MCforGN determines related genes based on their co-occurrence in MEDLINE abstracts. It employs both the standard centrality measures and Monte Carlo simulation to identify genetic networks and disease-gene associations.
  We evaluated the performance of our system using the common centrality measures across all approaches (i.e., Closeness, Betweenness, Degree). We report the precision of the top 10 ranked genes by each centrality measure and by each approach in Table 13. As can be seen from the table, The System performs well, and the results are both balanced and comparable with the other approaches. There are two main observations that can be seen from the table:
  - **The first observation** is that our system scored the best precision by closeness centrality measure, and this is an expected performance improvement from applying threshold ranking. Scoring the highest in the closeness measure is also an indication of the system’s ability to predict disease-related genes and the significance of using threshold ranking. In general, the closeness metric is the best metric to determine the *global* importance of a node in the network, whereas the degree and betweenness metrics can better determine the *local* importance of the node in the network. For example, in a network of criminals, each node represents a criminal. Using the degree and betweenness centrality would identify the immediate criminal leaders in the network. However, using the closeness metric would identify the main leader(s) of the whole criminal network (In our case, identify the main genes that are related to the disease).

**Table 13:** A comparison for the precision of the top 10 ranked genes by each centrality measure and by each approach

	Closeness	Betweenness	Degree
CGDA [14]	70	90	80
EDC-EDC [42]	77.3	86.4	82.8
MCforGN [43]	78	83	82
*Proposed system*	80	80	80
  - **The second observation** is that our system has comparable results with the other approaches, which not only indicates good performance, but it also shows the system can predict disease-related genes from gene interaction networks. Some of the genes that were predicted by the system were not found to be disease-related according to the benchmarks. These genes can still be good candidates for experimental verification because the benchmarks that were used are still under an ongoing effort of research. For example, our system has predicted 80% of prostate cancer genes correctly according to PGDB (recall Table 13). The remaining 20% of genes were not verified by PGDB. However, their relation to prostate cancer can be verified further by another benchmark or by working with a biologist to conduct an experimental test. Working with a biologist is one of the main directions that we would like to follow to evaluate our system.

## Conclusion

In this work, we presented a system for the identification of disease-gene associations. We used the initial set of seed genes known to be related to the disease to retrieve their neighbor genes from the human co-occurrence network generated by the system. Network analysis was then applied to the constructed subnetworks (disease-related networks) using a network analysis visualization tool. We applied closeness, betweenness, degree and eigenvector centrality measures to rank the genes in the subnetworks and to identify new candidate genes that could be linked directly to the diseases. In this study, we focus on studying cancer-related genes as cancer is one of the top 10 leading causes of death in the world. We evaluate the performance of the system by using disease-gene related benchmarks against the top 15 ranked genes. Degree and eigenvector centrality achieves the highest precisions for identifying breast, prostate, and lung cancer genes. According to one benchmark, betweenness and eigenvector centrality predicted correctly 100% of the breast-cancer-related genes. Our system predicted 80% of prostate-related genes using both closeness and eigenvector centrality. We also evaluated the system in terms of recall performance measures, and we report the percentage of initial seed genes that are retrieved among the top 15-20 ranked genes by each centrality measure.

One of the main directions that we would like to follow to evaluate our system, and show the significance of our work is through working with a biologist. Turning to a biologist to conduct an experimental test can help us verify the prediction genes. Some of the genes that were predicted by the system were not found to be disease-related according to the benchmarks we used. These genes, however, can still be good candidates for experimental verification because the benchmarks that were used are still under an ongoing effort of research.

There are few directions to consider for improving the results produced by the proposed system. The first is to increase the accuracy for predicting the connected and un-connected genes, as well as, the recall and precision. In this study, we only considered the primary names of genes (official gene symbol). Perhaps the use of gene names like synonyms, or gene numbers (referred to as Ordered Locus Names by UniProt [25]) could enhance the quality of performance as some authors refer to genes using alias names in the biomedical articles.

Another direction related to the information extraction component is to follow new structural linguistics principles and Natural Language Processing methods. For example, our system’s linguistic model does not consider the long distance relationship between genes or gene-GOterms as the algorithm looks at each sentence in the abstract at a time. In the future, we intend to investigate more descriptive linguistic theories and different NLP techniques to allow for a better extraction of the genes relation.

Another aspect to consider is the extension of the steps followed by this approach to further include the context of the study. The cancer type of study could be added as part of the extracted features, since improving the results of the system in constructing the network will directly be reflected in the identification of disease-gene associations. Towards the same directions, the set of abstracts chosen in this study could have affected the prediction accuracy. Therefore, for future work, we could take into account the full-text articles provided by reliable resources.

## Additional file

Additional file 1 Document containing the list of genes for each cancer type according to MalaCards and NCI’s GDC. (XLSX 35 kb)

## Abbreviations

BioNLP: Biological natural language processing
DGA: Disease-gene association
GO term: Gene Ontology term
NIH: The national institutes of health
NCCDPHP: National center for chronic disease prevention and health promotion
NLP: Natural language processing
OMIM: Online mendelian inheritance in man
PFP: Protein function prediction
PPIs: Protein-protein interactions
RBF: The Gaussian radial basis function
WKLR: Weighted kernel logistic regression
WLR: Weighted logistic regression

## References

[1] Centers for Disease Control and Prevention. Leading causes of death and numbers of deaths, by sex, race, and Hispanic origin: United States, 1980 and 2014 (Table 19). Health, United States, 2015. https://www.cdc.gov/nchs/data/hus/hus15.pdf. Accessed 22 Sept 2017.

[2] National Cancer Institute at the National Institutes of Health. Common Cancer Types. Atlanta; 2016. https://www.cancer.gov/types/common-cancers. Accessed 23 Aug 2017.

[3] American Cancer Society: Cancer Facts and Figures 2017. Atlanta American Cancer Society; 2017. https://www.cancer.org/research/cancer-facts-statistics/all-cancer-facts-figures/cancer-facts-figures-2017.html. Accessed 23 Aug 2017.

[4] Mohammad RM, Muqbil I, Lowe L, Yedjou C, Hsu H-Y, Lin L-T, Siegelin MD, Fimognari C, Kumar NB, Dou QP (2015). Broad targeting of resistance to apoptosis in cancer. Seminars in Cancer Biology.

[5] Feitelson MA, Arzumanyan A, Kulathinal RJ, Blain SW, Holcombe RF, Mahajna J, Marino M, Martinez-Chantar ML, Nawroth R, Sanchez-Garcia I (2015). Sustained proliferation in cancer: Mechanisms and novel therapeutic targets. Seminars in Cancer Biology.

[6] Pletscher-Frankild S, Palleja A, Tsafou K, Binder JX, Jensen LJ (2015). Diseases: Text mining and data integration of disease–gene associations. Methods.

[7] Khare R, Leaman R, Lu Z (2014). Accessing biomedical literature in the current information landscape. Biomed Lit Min.

[8] Mallory EK, Zhang C, Ré C, Altman RB (2015). Large-scale extraction of gene interactions from full-text literature using deepdive. Bioinformatics.

[9] Pandey G, Kumar V, Steinbach M (2006). Computational approaches for protein function prediction: A survey. Twin Cities Dep Comput Sci Eng Univ Minn.

[10] Entezari Heravi A (2015). Disease-gene association using genetic programming.

[11] Jung J-Y, DeLuca TF, Nelson TH, Wall DP (2013). A literature search tool for intelligent extraction of disease-associated genes. J Am Med Inform Assoc.

[12] Rebholz-Schuhmann D, Grabmüller C, Kavaliauskas S, Croset S, Woollard P, Backofen R, Filsell W, Clark D (2014). A case study: semantic integration of gene–disease associations for type 2 diabetes mellitus from literature and biomedical data resources. Drug Discov Today.

[13] Adamic LA, Wilkinson D, Huberman BA, Adar E (2002). A literature based method for identifying gene-disease connections. Bioinformatics Conference, 2002. Proceedings. IEEE Computer Society.

[14] Özgür A, Vu T, Erkan G, Radev DR (2008). Identifying gene-disease associations using centrality on a literature mined gene-interaction network. Bioinformatics.

[15] Quan C, Ren F. Gene–disease association extraction by text mining and network analysis. In: Proceedings of the 5th International Workshop on Health Text Mining and Information Analysis (Louhi)@ EACL: 2014. p. 63.

[16] Al-Mubaid H, Singh RK (2005). A new text mining approach for finding protein-to-disease associations. Am J Biochem Biotechnol.

[17] Hou W-J, Chen L-C, Lu C-S (2011). Identifying gene-disease associations using word proximity and similarity of gene ontology terms. Biomedical Engineering and Informatics (BMEI), 2011 4th International Conference On.

[18] Sun K, Gonçalves JP, Larminie C, Pržulj N (2014). Predicting disease associations via biological network analysis. BMC Bioinformatics.

[19] Zhu F, Patumcharoenpol P, Zhang C, Yang Y, Chan J, Meechai A, Vongsangnak W, Shen B (2013). Biomedical text mining and its applications in cancer research. J Biomed Inform.

[20] Topinka CM, Shyu C-R (2006). Predicting cancer interaction networks using text-mining and structure understanding. AMIA Annual Symposium Proceedings.

[21] Kim J, So S, Lee H-J, Park JC, Kim J-j, Lee H (2013). Digsee: disease gene search engine with evidence sentences (version cancer). Nucleic Acids Res.

[22] Maalouf M, Siddiqi M (2014). Weighted logistic regression for large-scale imbalanced and rare events data. Knowl-Based Syst.

[23] Al-Aamri A, Taha K, Al-Hammadi Y, Maalouf M, Homouz D. Constructing genetic networks using biomedical literature and rare event classification. Sci Rep. 2017; 7. 10.1038/s41598-017-16081-2 PMC5694017 29150626

[24] Maalouf M, Trafalis TB (2011). Robust weighted kernel logistic regression in imbalanced and rare events data. Comput Stat Data Anal.

[25] The universal protein resource (UniProt). http://www.uniprot.org/. Accessed 13 July 2016.

[26] Consortium GO (2004). The gene ontology (go) database and informatics resource. Nucleic Acids Res.

[27] Binns D, Dimmer E, Huntley R, Barrell D, O’donovan C, Apweiler R (2009). Quickgo: a web-based tool for gene ontology searching. Bioinformatics.

[28] Benson D, Boguski M, Lipman DJ, Ostell J (1990). The national center for biotechnology information. Genomics.

[29] Carpenter B (2007). Lingpipe for 99.99% recall of gene mentions. Proceedings of the Second BioCreative Challenge Evaluation Workshop.

[30] Maalouf M, Trafalis TB, Adrianto I (2011). Kernel logistic regression using truncated newton method. Comput Manag Sci.

[31] Maalouf M (2011). Logistic regression in data analysis: an overview. International Journal of Data Analysis Techniques and Strategies.

[32] Maalouf M, Humouz D, Kudlicki A (2014). Robust weighted kernel logistic regression to predict gene-gene regulatory association. IIE Annual Conference. Proceedings.

[33] Maalouf M, Homouz D (2014). Kernel ridge regression using truncated newton method. Knowl-Based Syst.

[34] Szklarczyk D, Franceschini A, Wyder S, Forslund K, Heller D, Huerta-Cepas J, Simonovic M, Roth A, Santos A, Tsafou KP (2014). String v10: protein–protein interaction networks, integrated over the tree of life. Nucleic Acids Res.

[35] Efron B, Tibshirani RJ (1994). An Introduction to the Bootstrap.

[36] Hamosh A, Scott AF, Amberger JS, Bocchini CA, McKusick VA (2005). Online mendelian inheritance in man (omim), a knowledgebase of human genes and genetic disorders. Nucleic Acids Res.

[37] Shannon P, Markiel A, Ozier O, Baliga NS, Wang JT, Ramage D, Amin N, Schwikowski B, Ideker T (2003). Cytoscape: a software environment for integrated models of biomolecular interaction networks. Genome Res.

[38] Rappaport N, Nativ N, Stelzer G, Twik M, Guan-Golan Y, Iny Stein T, Bahir I, Belinky F, Morrey CP, Safran M, et al. Malacards: an integrated compendium for diseases and their annotation. Database. 2013;2013. 10.1093/database/bat018 PMC3625956 23584832

[39] Rappaport N, Twik M, Plaschkes I, Nudel R, Iny Stein T, Levitt J, Gershoni M, Morrey CP, Safran M, Lancet D (2017). Malacards: an amalgamated human disease compendium with diverse clinical and genetic annotation and structured search. Nucleic Acids Res.

[40] The NCI’s Genomic Data Commons (GDC). https://gdc.cancer.gov. Accessed 12 Sept 2017.

[41] Futreal PA, Coin L, Marshall M, Down T, Hubbard T, Wooster R, Rahman N, Stratton MR (2004). A census of human cancer genes. Nat Rev Cancer.

[42] Taha K (2015). Extracting various classes of data from biological text using the concept of existence dependency. IEEE J Biomed Health Informat.

[43] Al-Dalky R, Taha K, Al Homouz D, Qasaimeh M (2016). Applying monte carlo simulation to biomedical literature to approximate genetic network. IEEE/ACM Trans Comput Biol Bioinforma.

[44] Li L-C, Zhao H, Shiina H, Kane CJ, Dahiya R (2003). Pgdb: a curated and integrated database of genes related to the prostate. Nucleic Acids Res.

